# Supplementary material for: Causal Relationship between Aging and Anorexia Nervosa: A White-Matter-Microstructure-Mediated Mendelian Randomization Analysis
Source: Biomedicines. 2024 Aug 16;12(8):1874. doi: 10.3390/biomedicines12081874 (PMC11351342; doi:10.3390/biomedicines12081874)
Supplement: Supplementary file 1 [file biomedicines-12-01874-s001.zip › Supplementary Figure/Figure S8/Leaveoneout plot of MR analysis for TL to ubm-b-1609.pdf]

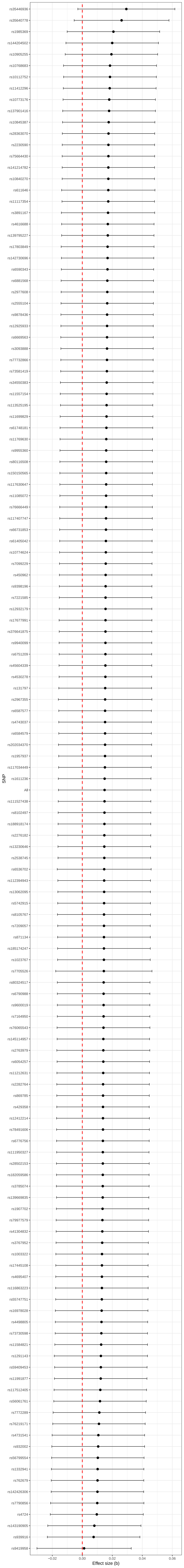

rs35446936
rs35640778
rs1985369
rs144204502
rs10905255
rs10768683
rs10112752
rs11412296
rs10773176
rs137901416
rs10845387
rs28363070
rs2230590
rs75664430
rs141214782
rs10840270
rs611646
rs11117354
rs3891167
rs4616688
rs139795227
rs17803849
rs142730696
rs6590343
rs6881568
rs2977608
rs2555104
rs9878438
rs12925933
rs6669563
rs3093888
rs77732866
rs73581419
rs34550383
rs11557154
rs113525195
rs11699829
rs61748181
rs11769630
rs9955360
rs80116508
rs150150565
rs117630647
rs11085072
rs76666449
rs117407747
rs66731853
rs61405042
rs10774624
rs7099229
rs450962
rs9398196
rs7221585
rs12932179
rs17677991
rs376641875
rs9940099
rs6751209
rs45604339
rs4530278
rs131797
rs2967355
rs6587577
rs4743037
rs6584579
rs202034370
rs1957937
rs117034449
rs1611236
All
rs111527438
rs8102497
rs188918174
rs2276182
rs13230646
rs2538745
rs6536702
rs112394943
rs13062095
rs5742915
rs8105767
rs7209057
rs871134
rs185174247
rs1023767
rs7705526
rs80324517
rs6790988
rs9600019
rs7164950
rs76065543
rs145114957
rs2763979
rs6054257
rs11212631
rs2282764
rs869785
rs429358
rs12412214
rs78491606
rs6776756
rs111950327
rs28502153
rs182059586
rs3785074
rs139669835
rs1907702
rs79977579
rs41304832
rs3767952
rs1003322
rs17445108
rs4695407
rs116863223
rs55747751
rs16978028
rs4498805
rs73730598
rs11584821
rs1291143
rs59409453
rs11991877
rs117512405
rs56061761
rs7772289
rs76219171
rs4731541
rs932002
rs56799554
rs1332941
rs782679
rs142426306
rs7780856
rs4724
rs143190905
rs939916
rs9419958
SNP
-0.02
0.00
0.02
0.04
0.06
Effect size (b)
